# Autochthonous West Nile Virus Infection Outbreak in Humans (Asti, Piedmont, Italy, August–October 2018) and Long-Term Sequelae Follow-Up

**DOI:** 10.3390/tropicalmed7080185

**Published:** 2022-08-16

**Authors:** Tommaso Lupia, Valentina Libanore, Silvia Corcione, Valentina Fornari, Barbara Rizzello, Roberta Bosio, Giacomo Stroffolini, Paolo Bigliano, Silvia Fontana, Francesca Patti, Maria Teresa Brusa, Maria Degioanni, Erika Concialdi, Anna Sara Navazio, Maurizio Penna, Francesco Giuseppe De Rosa

**Affiliations:** 1 Unit of Infectious Diseases, Cardinal Massaia Hospital, 14100 Asti, Italy; 2 Department of Medical Sciences, Infectious Diseases, University of Turin, 10124 Torino, Italy; 3 Unit of Laboratory Medicine and Microbiology, Cardinal Massaia Hospital, 14100 Asti, Italy

**Keywords:** West Nile virus, meningitis, encephalitis, outbreak

## Abstract

West Nile virus (WNV) infection is a reemerging zoonosis recently provoking significant outbreaks throughout Europe. During the summer of 2018, the number of WNV infections rose with a peak of new diagnoses of West Nile neuro-invasive disease (WNND). Most of the Italian cases were clustered in the Po River Valley. We present a case series of nine patients with WNV infection admitted to the Cardinal Massaia Hospital from 30 August 2018 to 1 October 2018. Demographic, immunovirological, clinical and therapeutic data are shown, and a report on clinical sequelae from the subsequent follow-up in patients with WNV and WNND. We showed the clinical, radiological and biochemical characteristics of WNV-infected patients. The risk factors and the clinical presentation of WNV in most patients in our case series were typical of that described in the literature, although, despite the high morbidity and mortality of WNND, we showed survival of 100% and long-term sequelae in only three patients. Environmental conditions may be essential in WNV outbreaks, and WNND can be clinically neurological multiform. Our long-lasting follow-up with clinical or radiological monitoring confirmed the morbidity of long-term neurological sequelae after WNND. Further studies are needed to investigate the epidemiology and physiopathology of bacterial superinfections after WNV infection.

## 1. Introduction

West Nile virus (WNV) is a mosquito-borne RNA virus belonging to the genus *Flavivirus* (family *Flaviviridae*) [1]. Since its discovery in Uganda in 1937, the WNV has been accounted responsible for thousands of deaths both in animals and in humans worldwide [2]. Birds are considered the main reservoir of WNV as they can develop a sufficiently high viraemia and cause the infection of a feeding mosquito [1,2]. The ornithophilic mosquitoes from the genus *Culex*, *Aedes* and *Ochlerotatus* are considered the main vectors since they can transmit the virus from an infected bird to another one through salivary gland secretions [1,2]. Humans and equids are dead-end hosts since their viraemia is insufficient to infect mosquitoes. Mosquitoes that feed on both birds (amplification hosts) and mammals (occasional hosts) are referred to as bridges [1]. Blood transfusion and transfusion/transplantation of substances of human origin (i.e., blood, organs or cells), percutaneous or conjunctival exposure in laboratories, or transplacental passage from mother to fetus are additional transmission routes among humans [2].

From the clinical point of view, more than 80% of WNV-infected individuals follow an asymptomatic course, whereas those who are symptomatic generally exhibit a mild flu-like syndrome (i.e., West Nile fever, WNF). Severe neuroinvasive disease (i.e., West Nile neuro-invasive disease, WNND) is observed in 1% of infected individuals or 10% of those symptomatic, particularly elderly chronically ill and immunocompromised subjects. The clinical presentation of WNND includes meningitis, encephalitis, and acute flaccid paralysis [2].

Albeit infrequent, WNND is a severe condition associated with significant morbidity and mortality which can lead to the death of the patient and although horse vaccines are currently available, no human vaccines or specific antiviral treatments have been to date licensed. Furthermore, the multiple routes of transmission and species involved result in a complex public health threat that requires a holistic approach for effective disease prevention and control. [3]

Often described as a reemerging disease, WNV numbers and geographic extension have increased, possibly due to climate change and its ecologic consequences [4] and, despite being an uncommon cause of meningoencephalitis, it has recently provoked significant outbreaks throughout Europe.

WNV infection is a zoonosis endemic in many parts of Europe. Between 2010 and 2018, 13 EU countries (Austria, Bulgaria, Croatia, Cyprus, Czechia, France, Greece, Hungary, Italy, Portugal, Romania, Slovenia and Spain) plus five EU enlargement countries (Albania, Montenegro, Serbia, Turkey and Kosovo) reported 3849 cases of human WNV infections [5]. For the majority of the countries, 2018 was the year when the highest number of cases was reported [5].

Particularly in Italy, 1145 WNV infection cases, including 487 WNND (42.5%), were notified between 2012 and 2020 [6,7]. The first reported WNND in Italy was described in Emilia-Romagna in September 2008 [6]. The number of reported cases peaked during 2018 for both new diagnoses and reported cases of WNND. Most of the reported new diagnoses of WNV infections were clustered in Northern Italy, and more precisely in areas belonging to Po River Valley, with only sporadic cases from Central Italy, Southern Italy, and Sardinia. When focusing on the region of origin, the majority of cases occurred in Emilia Romagna, followed by Veneto, Lombardy, Piedmont (9.7%), and Friuli-Venezia-Giulia [7].

We aimed to describe the clinical presentation, early outcomes and long-term follow-up of a recent outbreak of WNV infections that occurred in the Piedmont region (Italy) considering patients admitted at Cardinal Massaia Hospital, Asti, between August and October 2018.

## 2. Materials and Methods

### 2.1. Cohort and Samples

We included all suspected, and laboratory-confirmed cases of acute WNF and WNND admitted to the Cardinal Massaia Hospital from 30 August 2018 to 1 October 2018. A confirmed case of WNND was defined as having compatible neurological symptoms and the detection of anti-WNV immunoglobulin M (IgM) in a single serum sample or WNV RNA positive blood or urine sample detected by either PCR or real-time PCR. All patients with a suspected diagnosis of WNND were undergoing lumbar punctures for clinical reasons.

Demographic, immunovirological, clinical and therapeutic data were recorded as well as CSF characteristics. In addition, in the present study, we included all deaths that occurred within three months of diagnosis. Demographic and clinical data were available and extracted.

### 2.2. Viral and Immunological Measures

The presence of WNV RNA was tested by Real-Time reverse transcriptase Polymerase Chain Reaction (qRT-PCR), (Cobas^®^ TaqScreen West Nile Virus kit; Roche Molecular System, Branchburg, NJ, USA). In addition, IgM and IgG antibodies were detected against WNV in serum by enzyme-linked immunosorbent assay (ELISA; WNV IgM capture DxSelect ELISA and IgG DxSelect ELISA kits by Focus Diagnostics Inc., Cypress, CA, USA).

### 2.3. Statistical Analysis

We performed descriptive statistics on the entire study population and data were analyzed using standard statistical methods. Variables were described with median variables (interquartile ranges), absolute values and rates. Data analysis was performed using SPSS software for Mac (version 27.0. IBM Corp, Armonk, New York City, NY, USA). Graphs were created with SPSS and ppt.

## 3. Results

From August 2018 to October 2018, nine patients were hospitalized with WNV infection and 88.8% (*n* = 8) of nine with a neuroinvasive disease, WNND. Most hospitalized patients with WNV were males (5; Table 1) and caucasian, Italian (9) (Table 1).

The median age was 63 (interquartile range [IQR], 29–79). 6 (66.6%) had the presentation of meningitis, and the rest had presented with cerebellitis (1) and meningo-encephalitis (1). Furthermore, one case of meningitis was complicated by optic neuritis and the case of meningo-encephalitis was complicated by peripheral neuropathy (Table 1).

A total of 88.8% (*n* = 8) of nine patients hospitalized with WNV neuroinvasive disease had underlying conditions, including seven (77.7%) with vascular diseases, such as cerebral or peripheral vascular disorders, hypertension, 3 (33.3%) with cardiovascular diseases, and three (33.3%) with diabetes mellitus or onco-haematological diseases 3 (33.3%) (Table 1).

As detailed in Table 1, systemic symptoms such as fever, nausea and vomiting, headache, joint pain and fatigue were common in patients with WNV infection. In addition, confusion, meningism, aphasia, and apraxia, among neurological signs and symptoms, were the more frequently observed (Table 1).

**Figure 1:**
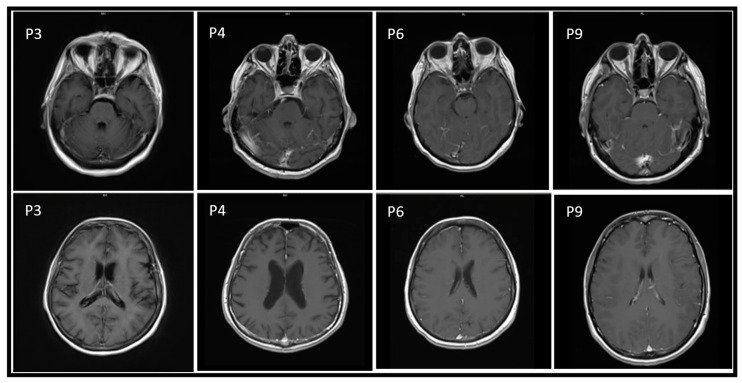
Main MR findings of patients. Axial contrast-enhanced image shows meningeal increased signal intensity accordingly to the clinical suspect of meningitis in patients 3, 4, 6 and 9 (respectively, P3, P4, P6 and P9).

Eight patients (88.8%) underwent MR, and abnormalities were shown in 62.5% (five), mostly with meningeal impregnation (Table 1, Figure 1).

A mild diffused meningeal contrast enhancement is the main radiologic finding in T1-weighted images in our population. As shown above, it mostly regards the cranial dome meninges, though it can be noticed in more caudal levels as well. Other possible MRI features are hyperintensity within deep brain and midbrain structures, and increased signal intensity in the mesial temporal structures in T2-WI/FLAIR. Moreover, high diffusion signals within the basal ganglia and disseminated throughout the white matter are frequently described in DWI scans.

In addition, eight patients underwent EEG and five patients (62.5%) showed abnormalities as detailed in Table 1. One patient with hypotonia and lower limb weakness underwent EMG showing peripheral neuropathy.

Most patients were treated with a course of intravenous acyclovir and antibiotic therapy until the definite diagnosis of WNND. One patient was treated with intravenous immunoglobulin (IVIG) and a patient needed anticonvulsant (Table 1).

The median hospital length of stay was 14 (4–27) days. The rate of survival was 100%, despite that seven patients were discharged directly to home and the rest needed a long-term ward stay before being discharged.

Three patients developed clinical and neurological sequelae and were strictly followed-up in the subsequent months with a period comprised between 5 to 18 months after discharge with clinical and radiological monitoring as detailed in Table 1.

All the patients undergo lumbar punctures due to neurological signs and symptoms. Cerebrospinal fluid (CSF) cell count showed a median of 59 (±63.6) white blood cells, primarily lymphocytes (86%), median proteinorrachia level was 74 (±24.7) and glycorrachia 82.1 (±40.2) with blood glucose/CSF glucose ratio as detailed in Table 2.

All the patients underwent WNV serological tests, and WNV-RNA counts on CSF, blood serum and urine. Respectively, WNV IgM were positive in seven (77.7%) patients and IgG in three (33.3%). Moreover, WNV-RNA was detectable in five (55.5%) urine and two (22.2%) blood samples. No patients had detectable WNV-RNA on CSF samples as detailed in Table 3.

During the hospitalization four patients (44.4%) presented with suspected superinfections: two bloodstream infections and two infections of the paranasal sinus and auricular region.

## 4. Discussion

The present study describes the first outbreak of WNV infection in humans in Asti, (Piedmont, Italy) during the summer of 2018. Many of the patients in this case series exhibited neuroinvasive clinical signs of WNV infection, possibly highlighting an overall underreporting of the circulating virus. WNND was multiform in our patients, with cases of meningitis, encephalitis, cerebellitis, peripheral neuropathy and optic neuritis. Patients with neurological sequelae at the time of discharge were followed up via clinical or radiological monitoring for up to 18 months after discharge.

Intensified and continuous spread of WNV across northern Italy has been observed since 2008, particularly in 2018, when the circulation of WNV increased in central and southern Europe [8]. In 2018, there were 595 confirmed human cases of WNV infection in Italy. Of these, 238 cases manifested as WNND, with 237 autochthonous cases distributed in six regions (Veneto, Emilia-Romagna, Lombardy, Piedmont, Sardinia and Friuli-Venezia Giulia) and one imported case [7,8]. Interestingly, over time we observed an East-West geographical shift across the Alps that is in line with the present report and available data [4,7].

On the basis of surveillance data available for Italy, most of the cases in 2018 were reported between August and October (about 178 cases, 77.7%) [7]. The outbreak in Asti, reported herein also occurred between August and October 2018. In our case series, there were more males than females in the WNV group (64.9% vs. 55.5%) and WNND group (75.0% vs. 62.5%), with a slight reduction in the number of males affected as compared with data presented by Riccò et al. [7]. Moreover, in the study by Riccò et al. [7], males accounted for approximately one-third of the total cases (*n* = 152, 31.2%), although the data on the gender of WNND cases were limited to 2012–2016.

Corresponding to the Italian Istituto Superiore di Sanità, in 2018, the transmission of the WNV in Italy and South-East Europe began earlier than in previous years [9]. On 16 June 2018, the first human case of confirmed infection occurred in our country, and, as of 5 September 2018, 365 confirmed cases of infection were reported. In particular, 148 patients were reported with manifestations of a neuro-invasive type, of which 19 deaths, 169 cases of fever and 48 infections in asymptomatic blood donors [9]. It is noteworthy that, at the European level, the highest increase compared to the previous transmission season was observed in Bulgaria (15-fold) followed by France (13.5-fold) and Italy (10.9-fold) [10].

According to 2012–2020 surveillance in Italy, only 25.1% (*n* = 122) of WNV cases were aged younger than 64 years. In the present case series, 55.5% (*n* = 5) of WNV cases were younger than 64 years (median age: 63 years). The median age of the patients with WNV in our study population (i.e., 63 years) is more similar to that reported in other European populations (i.e., 66 years) [5] than that reported previously in Italy (i.e., 75 years) [7].

We reported almost all cases of WNND, which could be the result of multiple biases, such as the recording only of cases admitted to hospital wards and of WNV infections that needed to be hospitalized due to the worse clinical presentation. The 2018 surveillance year was atypical because it recorded 53.3% of all 2012–2020 WNV infection cases and 47.0% of all WNND infections, irrespective of hospitalization status [7,8].

When it comes to the year of interest for this report (2018), taking a closer look to the figure, according to SEREMI regional notification system (“Servizio di riferimento Regionale di Epidemiologia per la sorveglianza, la prevenzione e il controllo delle malattie infettive”) [11], 66 WNV infections were recorded in 2018 in the Piedmont region, of which 56 were symptomatic (46 classified as WNND). Of those cases, 10 were diagnosed in the Asti province (1 WNV, 1 WNF, 8 WNND). Wrapping up, these data point to an evident mismatch between the notification system and the possible actual figure. In fact, if we assume that WNND cases are the peak of the iceberg, accounting for about 1% of WNV, it is possible to estimate about 900 WNV cases in the sole Asti province. That should raise attention on under-reporting from clinicians and expand efforts from veterinary sciences and epidemiology stakeholders, leading to broader collaboration in tackling this emerging problem from the one-health perspective.

Based on recent studies [4,12], environmental conditions play an essential role in WNV outbreaks, with such outbreaks linked to periods during and after seasons characterized by record-breaking heat and precipitation deficits, as occurred in Asti, Piedmont, Italy between July 2018 and October 2018, according to the Agenzia Regionale per la Protezione Ambientale (ARPA) reports [13]. Moirano et al. reported an association between precipitation and the incidence rate of WNND, particularly in the presence of a low amount of weekly total precipitation 2–3 weeks before the diagnosis of WNV infections [4,10]. Other reports showed a strong direct correlation between the incidence of WNND and environmental temperature [4,7,12].

In our case series, the risk factors for WNV infection were mostly typical of those described in the literature [2,3]. In a recent review, Yeung et al. [14] attempted to define risk factors associated with neuroinvasive disease [14]. According to their review, there is a consensus that older age, non-white race and male gender are independently statistically associated with WNND [14]. In our case series, the median age (63 years) was in line with European surveillance data. According to Yeung et al. [14], age is one of the main risk factors for WNND and the odds ratios for patients aged 60–69 years compared to younger counterparts ranged between 2.1 and 10.5. In their review, the most common clinical risk factors for developing WNND were diabetes, hypertension and malignancy, which were the same as those found in our case series, although whether these risk factors were statistically associated with WNND is unclear [14].

The clinical presentation of WNV in most patients in our case series was typical of that described in the literature [2,3]. However, unlike the literature, in which symptoms of meningitis/meningoencephalitis ranged in severity from mild to fatal, survival was 100% in our case series.

Of interest, we describe two uncommon cases of a middle-aged female with WNV cerebellitis and a young male with meningo-encephalitis complicated by optic neuritis. The first patient was admitted to our ward with dysarthria, dysmetria and dizziness. CT and MR were negative. EEG showed abnormal diffuse low activity, and CSF analysis showed an increased proteinorrachia and a low glicorrachia. The patient was treated with IVIG and supportive care. After discharge, the patient was followed up for eight months in our center. Neuroradiological exams remained negative but with a reliquated dysmetria. To our knowledge, only one other case of cerebellitis after WNV infection has been reported in a young child [15,16]. The second patient presented with a fever and headache, in addition to malaise, vomiting, diarrhoea, arthralgia and vision disturbance, manifesting as deteriorating visual acuity. An ophthalmologic evaluation revealed suspected optic neuritis that resolved over time without sequelae, with concomitant meningitis. To our knowledge, in the literature, optic neuritis complicating WNND has rarely been reported in a case report or case series [16,17].

During hospitalization, four patients presented with bacterial superinfections from blood-stream infections or sinus and otitis infections. To our knowledge, bacterial superinfections reported in the literature are uncommon after WNV infection, despite the fact that post-viral superinfections are frequently seen after tropical (i.e., Dengue virus, Yellow fever virus) and not strictly tropical viral infections (i.e., RSV, parainfluenza).

In this study, we report clinical sequelae and subsequent follow-up in patients with WNV and WNND. Patel et al. [18] published a systematic review of long-term sequelae after WNV infection. In our series, the long-term sequelae were dysmetria, cognitive impairment, motor impairment, dysphagia, ambulation difficulty and epilepsy. Interestingly, in research that compared the physical and functional prognoses of patients with WNND and those with WNF, individuals with WNND were found to have a worse prognosis overall [18]. Specifically, patients with WNND were more likely to experience persistent fatigue, muscle pains, limb weakness, chronic kidney illness, balance issues, ambulation difficulty, decreased activity and work absences [18].

There are several limitations to this study. First, this is a report of an outbreak that may not accurately reflect the general demographics of Italy. Second, the small sample size and the lack of a comparator group were other limitations. Furthermore, we acknowledge that viruses in general and particularly *Flaviviridae* present considerable immunological cross-reactivity, resulting in a troublesome serodiagnosis due to false positive results, we should stress that Tick-Borne Encephalitis, Zika, and Dengue are not endemic to the Asti province area in which our study was focused. Only a very low prevalence was found in a very different area of this Italian region.

In conclusion, we showed the clinical, radiological and biochemical characteristics of WNV-infected patients admitted in the summer of 2018 in Asti, Italy. We faced a high prevalence of WNND theoretically due to the environmental factors, type of population and epidemiological features of the 2018 season. However, despite the high morbidity and mortality of WNND, we showed survival of 100% and long-term sequelae in only three patients. Moreover, the long-lasting follow-up, up to 18 months after dismission, confirmed the morbidity of long-term sequelae after WNND and would require a more structured approach to all neuroinvasive diseases.

## Figures and Tables

**Table 1:** Clinical and diagnostic characteristics of patients admitted and follow-up data.

	Patient 1	Patient 2	Patient 3	Patient 4	Patient 5	Patient 6	Patient 7	Patient 8	Patient 9
Age	44	45	79	78	63	76	49	70	29
Sex	F	M	F	M	F	F	M	M	M
Month of Admission	August	August	August	September	September	September	September	September	October
Comorbidities	G6PDH deficit	CLL	NIDDM, hypertension, NHL	NHL	Hypertension	COPD	Hypertension	NIDDM	None
Clinical features	Fever, chills, headache, abdominal pain, vomiting	Fever, headache, malaise vomiting, diarrhoea, arthralgia, visual deficit	Fever, sleepiness, dyspnea	Fever, dizziness	Dysarthria, dysmetria, dizziness	Fever, paresthesia, ideomotor slowdown, photophobia, intention tremor	Fever, chills, vomiting, impaired consciuosness	Fever, chills, vomiting, impaired consciousness	Fever, dizziness, ear pain
Neurological involvement	No	Yes, Meningitis and Optic Neuritis	Yes, Meningitis	Yes, Meningitis	Yes, Cerebellitis	Yes, Meningo-encephalitis with peripheral neuropathy	Yes, Meningitis	Yes, Meningitis	Yes, Meningitis
CSF abnormalities	LP not performed	Yes	Yes	Yes	Yes	Yes	Yes	Yes	Yes
Laboratory abnormalities	Pancytopenia	Anaemia, increased CRP	Lymphocytosis, increased CRP	Anaemia, increased CRP and LDH	Low albumin and vitamine B12	Leucocytosis, increased CRP	Leucocytosis, hyperglycemia, increades CRP and LDH	Leucocytosis, increased CRP	Leucocytosis, prolonged aPTT
CT	NA	NA	Abnormality is not appreciated	Abnormality is not appreciated	Abnormality is not appreciated	Ischaemic sequelae in the bilateral middle cerebral artery territories	NA	Ischaemic sequelae in the left temporal lobe	NA
MR	NA	Negative	Meningeal inflammation	Meningeal inflammation	Negative	Meningeal inflammation	Meningeal inflammation	Gliosis	Meningeal inflammation
EEG	NA	Negative	Negative	Negative	Abnormal diffuse slow activity	Encephalitis pattern	Slow activity in posterior lobes	Abnormal diffuse slow activity	Epileptiform pattern on right hemisphere
EMG	NA	NA	NA	NA	NA	Peripheral Neuropathy	NA	NA	NA
Treatment	Symptomatic	Symptomatic	Acyclovir, antibiotic, symptomatic	Acyclovir, antibiotic, symptomatic	Acyclovir, antibiotic, IVIG, symptomatic	Acyclovir antibiotic, symptomatic	Acyclovir, antibiotic, symptomatic	Acyclovir, antibiotic, symptomatic	Acyclovir, antibiotic, anticonvulsivant, symptomatic
Length of stay (days)	4	8	15	14	8	13	12	27	14
Outcomes	Self-hospital discharge	Discharged	Discharged	Discharged	Transferred to long-term stay ward	Discharged	Discharged	Transferred to long-term care ward	Discharged
Neurological sequelae		No	No	No	Dysmetria	No	No	Cognitive and motor impairment, dysphagia	Epileptic disorders, difficulty ambulating
Clinical follow-up	No	No	No	No	FKT	No	No	FKT	ID + FKT
Diagnostic follow-up	No	No	No	CT (negative)	MR (unchanged)	No	No	CT (unchanged)	MR and EEG (post inflammatory damage)
End of Clinical or Diagnostic follow-up	NA	NA	NA	April 2019	December 2018	NA	NA	January 2021	February 2020

Abbreviations: CSF: cerebrospinal fluid; CT: computed tomography; MR: magnetic resonance; EEG: electroencephalography; EMG: electromyography; LP: lumbar puncture; NA: not available; CRP: C-Reactive Protein; LDH: Lactate Dehydrogenase; FKT: physiotherapy; CLL: Chronic Lymphocitic Leucemia; NHL: Non-Hodgkin Lymphoma: NIDDM: Non Insulin Dependant Diabetes Mellitus; COPD: Chronic Obstructive Pulmonary Diseases; IVIG: intravenous immunoglobulin.

**Table 2:** Cerebrospinal fluid analysis and chemical-physical characteristics.

	Patient 1	Patient 2	Patient 3	Patient 4	Patient 5	Patient 6	Patient 7	Patient 8	Patient 9
Macroscopic characteristics	NA	Clear, transparent	Clear, cloudy	Clear, transparent	Clear, transparent	Clear, transparent	Clear, transparent	Clear, transparent	Clear, transparent
RBC count [cells/mm^3^]	NA								
WBC count [cells/mm^3^]	NA	159	99	113	6	198	57	60	35
WBC subpopulation	NA	Mononuclear cells	Mononuclear cells	Mononuclear cells	Mononuclear cells	PMN cells, mononuclear cells	Mononuclear cells	Mononuclear cells	Mononuclear cells
Proteins [mg/dL]	NA	86	79	81	96	97	35	83	65
Glucose [mg/dL]	NA	59	83	53	59	58	170	110	50
CSF/serum glucose ratio	NA	0.6	0.6	0.4	0.4	0.4	0.4	0.6	0.4

Abbreviations: RBC: Red Blood Cell; WBC: White Blood Cell; PMN: polymorphonucleate; CSF: cerebrospinal fluid; NA: not available.

**Table 3:** Microbiological features of WNV infected patients.

	Patient 1	Patient 2	Patient 3	Patient 4	Patient 5	Patient 6	Patient 7	Patient 8	Patient 9
WNV IgM (serum)	+	+	+	−	+	+	+	+	−
WNV IgG (serum)	−	−	−	−	+	−	−	−	−
WNV IgM/IgG (CSF)	NA	NA	NA	NA	NA	NA	NA	NA	NA
WNV RNA (serum)	+	−	NA	+	−	+	−	−	−
WNV RNA (CSF)	NA	−	−	−	−	−	NA	−	−
WNV RNA (urine)	NA	NA	NA	+	−	+	+	+	+
CSF culture	NA	NA	−	−	−	−	−	−	−
Film-array (CSF)	−	NA	−	−	−	−	−	−	−
Superinfection/co-infections (Y/N)	N	N	Y	Y	Y	N	N	N	Y
Superinfection/co-infections (Type)	N	N	BSI (*S. hominis*)	BSI (*S. epidermidis*)	Sinus infection	N	N	N	Otitis

Abbreviations: CSF: cerebrospinal fluid; WNV: West Nile Virus; BSI: Blood-Stream Infections; NA: Not Available; +: positive; −: negative; N: No; Y: Yes.

## Data Availability

The data presented in this study are available on request from the corresponding author.
